# Conditional mutual inclusive information enables accurate quantification of associations in gene regulatory networks

**DOI:** 10.1093/nar/gku1315

**Published:** 2014-12-24

**Authors:** Xiujun Zhang, Juan Zhao, Jin-Kao Hao, Xing-Ming Zhao, Luonan Chen

**Affiliations:** 1 Key Laboratory of Systems Biology, Institute of Biochemistry and Cell Biology, Shanghai Institutes for Biological Sciences, Chinese Academy of Sciences, Shanghai 200031, China; 2 Department of Mathematics, Xinyang Normal University, Xinyang 464000, China; 3 School of Chemical and Biomedical Engineering, Nanyang Technological University, Singapore 637459, Singapore; 4 LERIA, Department of Computer Science, University of Angers, Angers 49045, France; 5 Department of Computer Science, School of Electronics and Information Engineering, Tongji University, Shanghai 201804, China; 6 Collaborative Research Center for Innovative Mathematical Modelling, Institute of Industrial Science, University of Tokyo, Tokyo 153-8505, Japan

## Abstract

Mutual information (MI), a quantity describing the nonlinear dependence between two random variables, has been widely used to construct gene regulatory networks (GRNs). Despite its good performance, MI cannot separate the direct regulations from indirect ones among genes. Although the conditional mutual information (CMI) is able to identify the direct regulations, it generally underestimates the regulation strength, i.e. it may result in false negatives when inferring gene regulations. In this work, to overcome the problems, we propose a novel concept, namely conditional mutual inclusive information (CMI2), to describe the regulations between genes. Furthermore, with CMI2, we develop a new approach, namely CMI2NI (CMI2-based network inference), for reverse-engineering GRNs. In CMI2NI, CMI2 is used to quantify the mutual information between two genes given a third one through calculating the Kullback–Leibler divergence between the postulated distributions of including and excluding the edge between the two genes. The benchmark results on the GRNs from DREAM challenge as well as the SOS DNA repair network in *Escherichia coli* demonstrate the superior performance of CMI2NI. Specifically, even for gene expression data with small sample size, CMI2NI can not only infer the correct topology of the regulation networks but also accurately quantify the regulation strength between genes. As a case study, CMI2NI was also used to reconstruct cancer-specific GRNs using gene expression data from The Cancer Genome Atlas (TCGA). CMI2NI is freely accessible at http://www.comp-sysbio.org/cmi2ni.

## INTRODUCTION

Identifying the causal regulations between genes is the key to understand the biological processes within cells. Despite the great efforts from the community, such as ENCODE (1) and modENCOD (2), untangling the comprehensive gene regulation networks (GRNs) is still a challenging task (3). With the increasingly accumulated high throughput data, many computational approaches have recently been developed to reconstruct GRNs (4–6). In general, these GRN inference approaches fall into two categories, i.e. model-based and machine learning-based approaches (7,8). In model-based methods, the chemical reactions of transcription and translation as well as other cellular processes are generally described with linear or nonlinear differential equations, where the parameters represent the causal strengths of the corresponding regulations. Popular methods in this category include singular value decomposition (9,10), network component analysis (11), multiple linear regression (12–14), and linear programming (15,16). In machine learning-based approaches, the regulations between genes are described by different indexes (i.e. causal association) (17,18), including Pearson correlation coefficient (19,20), Bayesian network (21), information theory-based mutual information (MI) (22–26) and conditional mutual information (CMI) (27,28).

Among those popular methods, the mutual information (MI) has been widely used to construct GRNs due to its capability of characterizing the nonlinear dependency between genes (23,29). Recent study shows that comparing with other approaches, MI is a natural way to equitably quantify statistical associations (30). Another advantage of the MI-based methods is their ability to deal with thousands of variables (genes) in the presence of the limited number of samples (14,31,32). Since MI describes the statistical dependencies between two variables, edge in GRN implies possible functional dependency between the two connected genes but not necessarily causal regulation. In other words, the edge detected by MI may be a functional or indirect regulation through one or more intermediaries instead of a direct (or physical) interaction between a transcription factor (TF) and a gene. Therefore, the mutual information overestimates the regulation relationships to some extent and fails to distinguish indirect regulators from direct ones, thereby leading to possible false positives during the inference of GRNs (28,33–35). Recently, the conditional mutual information (CMI) was proposed to infer the causal regulations between genes (36). As an extension of MI, CMI is able to separate the direct regulations from those indirect ones. CMI has also been used to detect the activity of TFs and miRNAs in transcriptional and post-transcriptional regulations (27,37). However, the theoretical analysis shows that CMI tends to underestimate the regulation strength in some cases due to its statistical feature (38–40).

In many cases, the real regulations between nodes in a GRN are obscured by the noise in the data. Therefore, most network inference methods perform poorly with a high false positive rate. To address this issue, Brarzel *et al*. (33) and Feizi *et al*. (35) described easily implemented methods for identifying and removing erroneous links, thereby producing more accurate networks. However, it turns out that both of the two methods are related with the method of partial correlation, which is the correlation between two variables predicted linearly from all other variables (34). Both of the two approaches need to inverse the correlation matrix to achieve the result and they are only different in scaling the inverse correlation matrix. Well known to us, it is difficult to process the inverse of correlation matrix when large scale variables but with small samples are given (41). Hence, approximate methods used by the two approaches to achieve the inverse of correlation matrix will destroy the performance of network inference in many cases.

In this work, to overcome those problems, we propose a concept based on a new measure of causal strength (36), i.e. CMI2 (conditional mutual inclusive information), to quantify the causal associations between variables. To infer GRNs, a new algorithm, namely CMI2NI, is developed by combining CMI2 with path consistency (PC) algorithm. CMI2NI, which alleviates both the overestimation problem of MI and the underestimation problem of CMI based on information theory, gives a quantitative measurement of causal associations between two genes. With the hypothesis of Gaussian distribution for gene expression data, CMI2 can be calculated by a concise formula involving the covariance matrices of the related gene expression profiles. The proposed network inference method CMI2NI can not only accurately quantify causal associations but also reconstruct the correct topological structures of biological networks even with a small number of samples. The experimental results on the benchmark GRNs from DREAM challenge and the widely used SOS DNA repair network in *Escherichia coli* demonstrated the effectiveness of our CMI2NI. As a case study, CMI2NI was applied to reconstruct cancer-specific GRNs based on gene expression data from The Cancer Genome Atlas (TCGA), where the GRNs provide a global view of the regulatory circuit of the cancer genes.

## MATERIALS AND METHODS

### (Conditional) mutual information

Recently, MI and CMI have been widely used to reconstruct GRNs due to their advantages in measuring dependency between variables. In general, the gene expression data can be described as vectors, in which the elements denote the expression values of genes under different conditions. MI measuring the dependency between two variables (genes) *X* and *Y* can be defined as below (42),

(1) \begin{equation*} {\rm MI}(X,Y) = \sum\limits_{x \in X,\;y \in Y} {p(x,y)\log \frac{{p(x,y)}}{{p(x)p(y)}}} , \end{equation*}

where *p*(*x, y*) is the joint probability distribution of *X* and *Y*, and *p*(*x*) and *p*(*y*) are the marginal probability distributions of *X* and *Y*, respectively. With the widely adopted hypothesis of Gaussian distribution for gene expression data, formula (1) can be easily calculated using the following equivalent formula (43)

(2) \begin{equation*} {\rm MI}(X,Y) = \frac{1}{2}\log \frac{{|C(X)| \cdot |C(Y)|}}{{|C(X,Y)|}}, \end{equation*}

where *C* is the covariance matrix of variables and $\left| {\; \cdot \;} \right|$ is the determinant of matrix *C*. If variables (genes) *X* and *Y* are independent of each other, clearly MI(*X, Y*) = 0.

On the other hand, CMI measures conditional dependency between two variables (genes) given other variable(s) (gene(s)). The CMI of variables *X* and *Y* given *Z* is defined as

(3) \begin{equation*} {\rm CMI}(X,Y|Z){=}\sum\limits_{x \in X,\,\;y \in Y,\;z \in Z} {p(x,y,z)\log \frac{{p(x,y|z)}}{{p(x|z)p(y|z)}}} , \end{equation*}

where $p(x,y|z), p(x|z)$ and $p(y|z)$ are conditional probability distributions. Similarly, under the assumption of Gaussian distributions (28), formula (3) equals to

(4) \begin{equation*} {\rm CMI}(X,Y|Z) = \frac{1}{2}\log \frac{{|C(X,Z)| \cdot |C(Y,Z)|}}{{|C(Z)| \cdot |C(X,Y,Z)|}}, \end{equation*}

which is efficient to calculate CMI between two variables *X* and *Y* given one or more variables *Z*. For example, if the conditional variable *Z* = (*Z*_1_, *Z*_2_) is composed of two variables (genes) *Z*_1_ and *Z*_2_, we get the second-order CMI.

### Kullback–Leibler divergence-based casual strength measure

Recently, to accurately measure the causal strength between two genes, a measure based on Kullback–Leibler (KL) divergence was proposed (39). Before giving the definition of the measure, the interventional probability is described firstly. In a directed acyclic graph (DAG), if variable *Y* is regulated by variable *X* both directly and indirectly through variable *Z*, the interventional probability of moving the link from *X* to *Y* is defined as

(5) \begin{equation*} P_{X \to Y} (x,y,z) = P(x,z)\sum\limits_x {P(y|z,x)P(x)} , \end{equation*}

where $P(y|z,x)$ is a conditional probability distribution of *Y* given *Z* and *X*.

For the three variables mentioned above, the casual strength, i.e. regulation strength of the arrow from *X* to *Y* is defined (39) as

(6) \begin{equation*} C_{X \to Y} (X;Y|Z) = D_{{\rm KL}} (P(X,Y,Z)||P_{X \to Y} (X,Y,Z)), \end{equation*}

where $P(X,Y,Z)$ is the joint probability distribution of *X, Y* and *Z*, $P_{X \to Y} (X,Y,Z)$ is the interventional probability distribution of *X, Y* and *Z* for removing arrow $X \to Y$, and $D_{{\rm KL}} (P||P_{X \to Y} )$ is KL-divergence from $P(X,Y,Z)$ to $P_{X \to Y} (X,Y,Z)$. With above definition, measure $C_{X \to Y} (X;Y|Z)$ is unsymmetrical.

### Conditional mutual inclusive information for association measure

**Figure 1.**
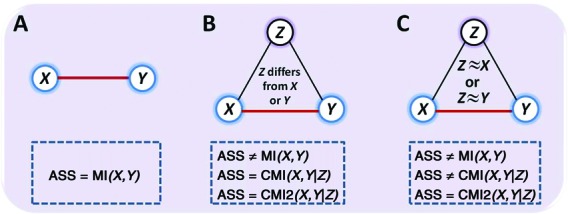
Quantification of association (ASS) by CMI2. (**A**) The association between two variables (genes) *X* and *Y* in a two-node network measured by CMI2 is equal to MI and CMI. (**B**) The association between two variables (genes) *X* and *Y* in a triple-node network measured by CMI2 is equal to CMI. (**C**) CMI can exclude the indirect information from direct information, but it underestimates the association between *X* and *Y* when the expression level of *Z* is near or equal to the expression level of *X* or *Y*. In this case, CMI2 can accurately measure the association prior to both MI and CMI.

Among the most popular association measures, MI tends to overestimate the regulation strengths between genes (false-positive problem), while CMI tends to underestimate the strengths (false-negative problem). As shown in Figure 1, MI can correctly quantify the regulation strength between genes *X* and *Y* for the case of Figure 1A but fails to quantify the association between genes *X* and *Y* for the case in Figure 1B due to the indirect regulation mediated by gene *Z*, i.e. overestimate the strength. Although CMI can successfully quantify the indirect regulation, it fails when the expression level of gene *Z* is near or equal to that of *X* (or *Y*), where the Pearson correlation coefficient between *Z* and *X* (or *Y*) is near or equal to 1. In that case as shown in Figure 1C, CMI will underestimate the regulation strength because the CMI value between genes *X* and *Y* given *Z* is near or equal to zero, which is actually incorrect. The newly proposed measure $C_{X \to Y} (X;Y|Z)$ tried to address this issue by calculating the relative entropy distance. However, it can only partially address the issue (see Supplementary Data). In addition, it is difficult to put it into practice for large-scale network inference because of the prior information requirement of network directions (38,39,44).

In this work, to overcome the problems discussed above, we proposed an effective unbiased measure based on the causal strength (39), named CMI2, to quantify causal associations between genes. CMI2 is an association measure using the inclusive information, i.e. entropy relative distance between the postulated edge-existence distribution and edge-non-existence distribution. Next, we describe the definitions of CMI2.

In a DAG, if variable *Y* is regulated by variable *X* both directly and indirectly through variable *Z*, the association between *X* and *Y* is defined as

(7) \begin{equation*} {\rm CMI}2(X,Y|Z){=}{{\left( {D_{{\rm KL}} (P||P_{X \to Y} ){+}D_{KL} (P||P_{Y \to X} )} \right)}/ 2}, \end{equation*}

where $P = P(X,Y,Z)$ is the joint probability distribution of *X, Y* and *Z*, $P_{X \to Y} = P_{X \to Y} (X,Y,Z)$ and $P_{Y \to X} = P_{Y \to X} (X,Y,Z)$ are the interventional probability distributions of *X, Y* and *Z* for removing edges $X \to Y$ and $Y \to X$, respectively. $D_{{\rm KL}} (P||P_{X \to Y} )$ and $D_{{\rm KL}} (P||P_{Y \to X} )$ are KL-divergences from *P* to$P_{X \to Y}$ and $P_{Y \to X}$. Similar to CMI, CMI2 has an order number $|Z|$, i.e. the number of conditional variables *Z*, and MI can be regarded as zero-order CMI2.

The above quantity can be decomposed into three terms. One of them is CMI and another two are non-negative terms. The decomposition can be derived from the theoretical result as follows. For the three variables defined above, ${\rm CMI}2(X;Y|Z)$ between variables *X* and *Y* given *Z* can be decomposed into

(8) \begin{equation*} \begin{array}{*{20}l} {{\rm CMI}2(X;Y|Z) = {\rm CMI}(X;Y|Z) + {\textstyle{1 \over 2}}D_{{\rm KL}} (P(Y|Z)} \\ {||P_{X \to Y} (Y|Z)) + {\textstyle{1 \over 2}}D_{{\rm KL}} (P(X|Z)||P_{Y \to X} (X|Z)).} \\ \end{array} \end{equation*}

The proof of the above result can be found in the Supplementary Data. Equation (8) states that CMI2 is equal to CMI if the second and third terms of Equation (8) are zero, i.e. both *X* and *Y* are independent with *Z*. Since the KL-divergence is non-negative, CMI2 between *X* and *Y* given *Z* is no less than CMI between *X* and *Y* given *Z*.

Equation (8) also states that CMI and $C_{X \to Y} (X;Y|Z) = {\rm CMI}(X;Y|Z) + D_{{\rm KL}} (P(Y|Z)||P_{X \to Y} (Y|Z))$ underestimate the association strength. For instance, given that *Y* and *Z* are almost identical, we can get ${\rm CMI}(X;Y|Z) \approx 0$ and $D_{{\rm KL}} (P(Y|Z)||P_{X \to Y} (Y|Z)) \approx 0)$. In other words, strong dependency between *X* and *Z* makes the influence of cause *Y* almost invisible when looking at ${\rm CMI}(X;Y|Z)$ and $D_{{\rm KL}} (P(Y|Z)||P_{X \to Y} (Y|Z))$. Therefore, The third term in Equation (8) corrects the underestimation. Similarly, if *X* and *Z* are similar, The second term in Equation (8) will correct the underestimation.

### Computation of CMI2

As described above, CMI2 can be determined by computing the (joint) probabilities of genes *X, Y* and *Z*, which can be estimated with kernel density estimator to construct the probability density functions based on gene expression data (23,27,45). In this work, to efficiently estimate CMI2, we assumed that the gene expression profiles follow a multivariate Gaussian distribution, which has been widely accepted and proved to be reasonable (43,46). Here, to approximate the Gaussian distribution, the log-transformation of gene expression data was adopted. According to the definition of KL-divergence, ${\rm CMI}2(X;Y|Z)$ can be rewritten as

(9) \begin{equation*} \begin{array}{*{20}l} {{\rm CMI}2(X;Y|Z) = } \\ {\sum\limits_{x,y,z} {P(x,y,z)\ln \frac{{P(x,y,z)}}{{P(x,z)\sum\limits_x {P(y|z,x)P(x)} + P(y,z)\sum\limits_y {P(x|z,y)P(y)} }}} ,} \\ \end{array} \end{equation*}

where $P(y|z,x)$ and $P(x|z,y)$ are the conditional probabilities.

With the hypothesis of Gaussian distribution, CMI2 can be calculated based on the following result.

**Theorem 1**. Let *X* and *Y* be 1-dimension variables, *Z* is a $n_z (n_z \ge 1)$-dimension variable, and *X, Y* and *Z* follow Gaussian distribution. Then

(10) \begin{eqnarray*} &&{\rm CMI}2(X;Y|Z) = \nonumber \\ &&\frac{1}{4}\left( {{\rm tr}(C^{ - 1} \Sigma ) + {\rm tr}(\tilde C^{ - 1} \tilde \Sigma ){\rm + }\ln C_0 + \ln \tilde C_0 - 2n} \right), \end{eqnarray*}

where

\begin{equation*} \begin{array}{*{20}l} {n = n_z + 2,C_0 = \rho _{xx} \left( {\left( {\Sigma ^{ - 1} } \right)_{xx} - \left( {\Sigma _1^{ - 1} } \right)_{xx} + \rho _{xx} ^{ - 1} } \right),} \\ {\tilde C_0 = \rho _{yy} \left( {\left( {\tilde \Sigma ^{ - 1} } \right)_{yy} - \left( {\tilde \Sigma _1^{ - 1} } \right)_{yy} + \rho _{yy} ^{ - 1} } \right),} \\ \end{array} \end{equation*}

\begin{equation*} C = \left( {\begin{array}{*{20}l} {C_{xx} } & {C_{xy} } & {C_{xz} } \\ {C_{xy} } & {C_{yy} } & {C_{yz} } \\ {C_{xz}^T } & {C_{yz}^T } & {C_{zz} } \\ \end{array}} \right)^{ - 1} ,\;\tilde C = \left( {\begin{array}{*{20}l} {\tilde C_{yy} } & {\tilde C_{yx} } & {\tilde C_{yz} } \\ {\tilde C_{yx} } & {\tilde C_{xx} } & {\tilde C_{xz} } \\ {\tilde C_{yz}^T } & {\tilde C_{xz}^T } & {\tilde C_{zz} } \\ \end{array}} \right)^{ - 1} , \end{equation*}

\begin{equation*} \begin{array}{*{20}l} {C_{xx} = \left( {\Sigma _1^{ - 1} } \right)_{xx} ,C_{xy} = 0,C_{xz} = \left( {\Sigma _1^{ - 1} } \right)_{xz} ,} \\ {C_{yy} = \left( {\Sigma ^{ - 1} } \right)_{yy} - \left( {\Sigma ^{ - 1} } \right)^2 _{xy} \left( {\left( {\Sigma ^{ - 1} } \right)_{xx} - \left( {\Sigma _1^{ - 1} } \right)_{xx} + \rho _{xx} ^{ - 1} } \right)^{ - 1} ,} \\ {C_{yz} = \left( {\Sigma ^{ - 1} } \right)_{yz} - \left( {\Sigma ^{ - 1} } \right)_{xy} \left( {\left( {\Sigma ^{ - 1} } \right)_{xx} - \left( {\Sigma _1^{ - 1} } \right)_{xx} + \rho _{xx} ^{ - 1} } \right)^{ - 1} \left( {\left( {\Sigma ^{ - 1} } \right)_{xz} - \left( {\Sigma _1^{ - 1} } \right)_{xz} } \right),} \\ {C_{zz} = \left( {\Sigma ^{ - 1} } \right)_{zz} - \left( {\left( {\Sigma ^{ - 1} } \right)_{xx} - \left( {\Sigma _1^{ - 1} } \right)_{xx} + \rho _{xx} ^{ - 1} } \right)^{ - 1} \left( {\left( {\Sigma ^{ - 1} } \right)_{xz} ^T - \left( {\Sigma _1^{ - 1} } \right)_{xz} ^T } \right)} \\ {\left( {\left( {\Sigma ^{ - 1} } \right)_{xz} - \left( {\Sigma _1^{ - 1} } \right)_{xz} } \right),} \\ \end{array} \end{equation*}

\begin{equation*} \begin{array}{*{20}l} {\tilde C_{yy} = \left( {\tilde \Sigma _1^{ - 1} } \right)_{yy} ,\tilde C_{yx} = 0,\tilde C_{yz} = \left( {\tilde \Sigma _1^{ - 1} } \right)_{yz} ,} \\ {\tilde C_{xx} = \left( {\tilde \Sigma ^{ - 1} } \right)_{xx} - \left( {\tilde \Sigma ^{ - 1} } \right)^2 _{yx} \left( {\left( {\tilde \Sigma ^{ - 1} } \right)_{yy} - \left( {\tilde \Sigma _1^{ - 1} } \right)_{yy} + \rho _{yy} ^{ - 1} } \right),} \\ {\tilde C_{xz} = \left( {\tilde \Sigma ^{ - 1} } \right)_{xz} - \left( {\tilde \Sigma ^{ - 1} } \right)_{yx} \left( {\left( {\tilde \Sigma ^{ - 1} } \right)_{yy} - \left( {\tilde \Sigma _1^{ - 1} } \right)_{yy} + \rho _{yy} ^{ - 1} } \right)^{ - 1} \left( {\left( {\tilde \Sigma ^{ - 1} } \right)_{yz} - \left( {\tilde \Sigma _1^{ - 1} } \right)_{yz} } \right),} \\ {\tilde C_{zz} = \left( {\tilde \Sigma ^{ - 1} } \right)_{zz} - \left( {\left( {\tilde \Sigma ^{ - 1} } \right)_{yy} - \left( {\tilde \Sigma _1^{ - 1} } \right)_{yy} + \rho _{yy} ^{ - 1} } \right)^{ - 1} \left( {\left( {\tilde \Sigma ^{ - 1} } \right)_{yz} ^T - \left( {\tilde \Sigma _1^{ - 1} } \right)_{yz} ^T } \right)} \\ {\left( {\left( {\tilde \Sigma ^{ - 1} } \right)_{yz} - \left( {\tilde \Sigma _1^{ - 1} } \right)_{yz} } \right),} \\ \end{array} \end{equation*}

\begin{equation*} \begin{array}{*{20}l} {\Sigma _1 = \left( {\begin{array}{*{20}l} {\rho _{xx} } & {\rho _{xz} } \\ {\rho _{xz} } & {\rho _{zz} } \\ \end{array}} \right){\rm ,}\tilde \Sigma _1 = \left( {\begin{array}{*{20}l} {\rho _{yy} } & {\rho _{yz} } \\ {\rho _{yz} } & {\rho _{zz} } \\ \end{array}} \right){\rm ,}} \\ {\sum = \left( {\begin{array}{*{20}l} {\rho _{xx} } & {\rho _{xy} } & {\rho _{xz} } \\ {\rho _{xy} } & {\rho _{yy} } & {\rho _{yz} } \\ {\rho _{xz} } & {\rho _{yz} } & {\rho _{zz} } \\ \end{array}} \right),\tilde \sum = \left( {\begin{array}{*{20}l} {\rho _{yy} } & {\rho _{yx} } & {\rho _{yz} } \\ {\rho _{yx} } & {\rho _{xx} } & {\rho _{xz} } \\ {\rho _{yz} } & {\rho _{xz} } & {\rho _{zz} } \\ \end{array}} \right){\rm .}} \\ \end{array} \end{equation*}

The proof of Theorem 1 can be found in the Supplementary Data. With Equation (10), the CMI2 can be calculated in a very efficient way with the general hypothesis of Gaussian distribution underlying gene expression data.

### Path consistency algorithm

The path consistency (PC) algorithm is a widely used algorithm in graph theory and has been used for the reconstruction of gene regulation network. By gradually removing redundant edges in a network based on the conditional dependency between the nodes, PC-algorithm can construct a network with sparse topological structure. In particular, PC-algorithm is specifically useful for the sparse and scale-free networks, such as biological networks including GRNs. The PC-algorithm has been utilized for GRN inference by popular approaches, such as pcalg (36) and PCA-CMI (28), where partial correlation coefficient (PCC) and CMI were respectively used by pcalg and PCA-CMI to quantify the regulation strength.

### CMI2NI: GRN inference method based on CMI2

Given an expression dataset with *n* genes and *m* samples, we developed a novel algorithm, called CMI2NI, to infer its underlying GRN. In CMI2NI, after obtaining MI and CMI2 with Equations (2) and (10), the PC algorithm was then used to remove the (conditional) indirect regulations from the clique/complete graph. GRN inference will be performed by removing those edges without strong causal regulations recursively until there is no change in the network topology, e.g. at *L*th-order CMI2. CMI2NI improves the resulted GRN through accurately quantifying the causal regulation strength with CMI2.

**Figure 2.**
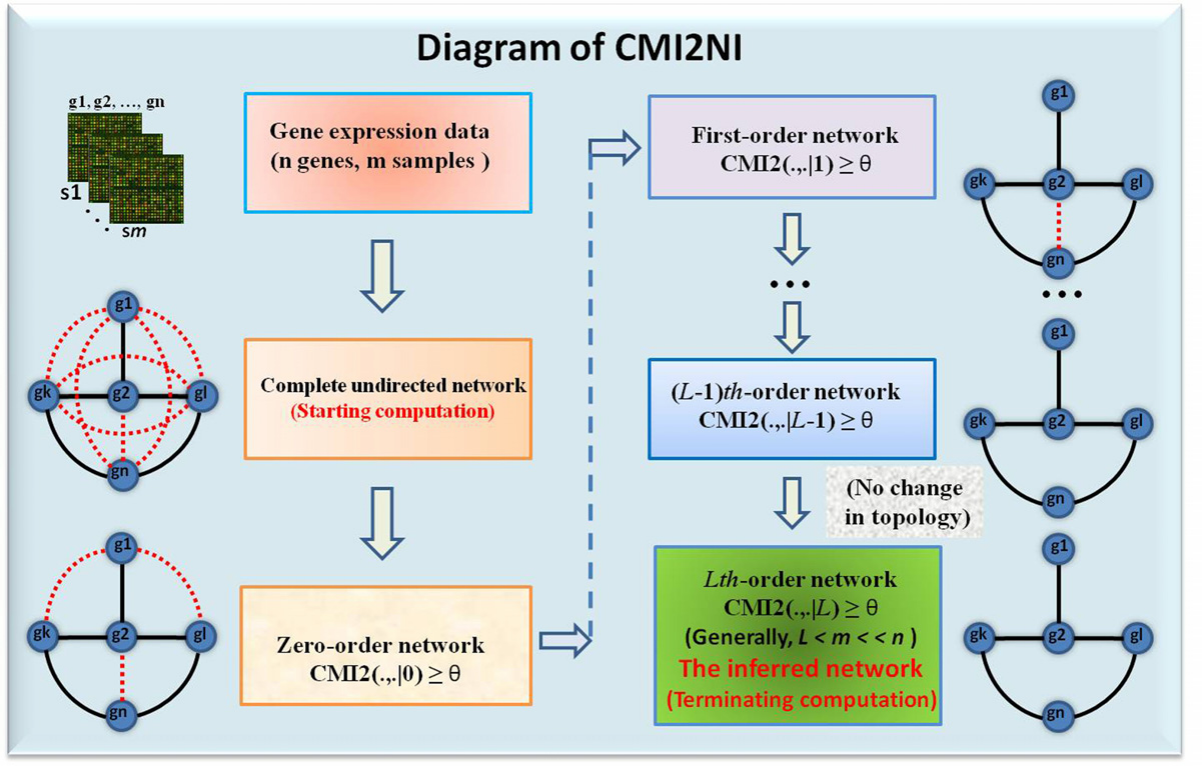
The diagram of CMI2NI. In the figure, ***g***_*i*_,*i* = 1, 2, …, *n* represent gene *i*, ***s****_j_, j* = 1, 2, …, *m*, represent sample *j*, and CMI2(.,. $|L$), *L* = 0, 1, …, *n* − 2, represents the *L*th-order CMI2. A pair of genes are regarded as independent if their CMI2 is equal to zero or below a given threshold. The black solid and red doted lines in the network represent direct and indirect regulations, respectively. The algorithm will terminate until there is no change between the (*L* − 1)th and *L*th-order networks, and the resultant network will be taken as the output of CMI2NI. Generally, we can obtain the network with a few iterations, i.e. *L < m << n*.

Figure 2 depicts the diagram of CMI2NI with details described as follows. Firstly, we generated a complete connected graph according to the number of genes. Secondly, for an adjacent gene pair *i* and *j*, we computed the MI between them. If the gene pair *i* and *j* has low or zero MI, we deleted the edge between genes *i* and *j*. Thirdly, for an adjacent gene pair *i* and *j*, we calculated the first-order CMI2 given another gene *z* that is neighbors of *i* and *j*. If the gene pair *i* and *j* has low or zero CMI2, we deleted the edge between them. Subsequently, the higher order CMI2 was calculated for a gene pair until there were no further changes in the network topology. The detailed algorithm for inferring a GRN was described in algorithm **CMI2NI**.

To reduce computational complexity but not sacrifice the accuracy for detecting the true regulatory interactions, we adopted an optimal strategy to select *L* genes from *T* adjacent genes for randomly selected gene pair *i* and *j*, which also ensures the local optimality of the algorithm. For example, suppose that there are *T* (*T* ≥ 1) genes which are adjacent with both genes *i* and *j*. When constructing the *L*th-order (*L* $ \le$ *T*) network, all the *L*th-order CMIs for the possible combinations of *L* conditional genes from *T* genes are computed and the maximal one or the geometric mean of them is selected to decide the existence of regulation. Generally, after a few number of iterations *L*, the computation will terminate due to no change on the network topology, i.e. *L < m << n*. In other words, we can obtain the network without resorting to any approximations in the computational procedure, as indicated in the CMI2NI algorithm and also Figure 2. Theoretically, for a network with *n* genes or molecules (e.g. *n* = 20 000 genes), it requires at least *n* independent samples to derive their direct regulations due to the computation of CMI2(.,.|*n* − 2), which clearly is not available for most of real cases. However, if the algorithm is converged to an *L*th-order network, it actually requires only *L* + 2 independent samples due to CMI2(.,.|*L*). In other words, CMI2NI can infer the network without any approximation in the above process even with a small number of samples, i.e. *L* + 2 independent samples when converged to an *L*th-order network, where *L* is usually between 3 and 5 for many real networks or datasets, far less than *n*. Thus, comparing to the traditional requirement of *n* independent samples for computing all statistic dependency among variables, our algorithm can obtain the network without the approximation with a small number of samples *L* + 2.

**Table:** 

**Algorithm (CMI2NI)**
**Input:**
Gene expression matrix ***A***,
Parameter for dependence threshold *θ*.
**Output:**
Inferred gene network ***G***,
Order of inferred network *L*.
**Step-1**. Initialization. Generate the complete connected network ***G_0_*** for all genes (i.e. the clique graph of all genes). Set $L: = - 1$.
**Step-2**.$L: = L + 1$; For a nonzero edge $G_0 (i,j) \ne 0$, select adjacent genes connected with both genes *i* and *j*. Compute the number *T* of the adjacent genes (not including genes *i* and *j*) .
**Step-3**. set $G : = G_0$. If $T < L$, stop. If $T \ge L$, select out *L* genes from these *T* genes and let them as $K = [k_1 , \cdots ,k_L ]$. The number of all selections for ***K*** is $C_T^L$. Compute the *L*th-order ${\rm CMI}2(i,j|K)$ for all $C_T^L$ selections, and choose the maximal one denoting as ${\rm CMI}2_{\max } (i,j|K)$. If ${\rm CMI}2_{\max } (i,j|K) < \theta$, set $G(i,j) = 0$.
**Step-4**. If $G = G_0$, stop; If $G = G_0$, set $G_0 : = G$ and return to Step-2.

Sometimes there is no need to run the algorithm with high order networks, so we set a parameter for deciding the maximal order of the network in the software to terminate the algorithm according to the user's need. More importantly, this will greatly reduce the computational complexity. The MATLAB implementation of the algorithm described above with detailed tutorials are freely available at http://www.comp-sysbio.org/cmi2ni.

### Datasets

In order to validate our method, CMI2NI was applied to simulation dataset as well as a real gene expression dataset. As for simulation data, the method was tested on the widely used reference network in *Yeast* with synthetic nonlinear expression data from DREAM challenge (4). As for real gene expression data, we applied our method to the well-known SOS DNA repair network with the experimental dataset in *E. coli* (47,48).

### Metrics for evaluation

The performance of the proposed method was evaluated by the following measures, i.e. sensitivity (SN) or true positive rate (TPR), false positive rate (FPR), positive predictive value (PPV), accuracy (ACC) and Matthews coefficient constant (MCC). Mathematically, they are defined as

\begin{equation*} {\rm TPR} = {\rm TP}/({\rm TP} + {\rm FN}), \end{equation*}

\begin{equation*} {\rm FPR} = {\rm FP}/({\rm FP} + {\rm TN}), \end{equation*}

\begin{equation*} {\rm PPV} = {\rm TP}/({\rm TP} + {\rm FP}), \end{equation*}

\begin{equation*} {\rm ACC} = ({\rm TP} + {\rm TN})/({\rm TP} + {\rm FP} + {\rm TN} + {\rm FN}), \end{equation*}

\begin{equation*} \begin{array}{*{20}l} {{\rm MCC} = } \\ {{{({\rm TP} \cdot {\rm TN}{-}{\rm FP} \cdot {\rm FN)}} /{\sqrt {({\rm TP}{+}{\rm FP})({\rm TP}{+}{\rm FN})({\rm TN}{+}{\rm FP})({\rm TN}{+}{\rm FN})} }},} \\ \end{array} \end{equation*}

where TP, FP, TN and FN are the numbers of true positives, false positives, true negatives and false negatives, respectively. TPR and FPR are also used to plot the receiver operating characteristic (ROC) curves and the area under ROC curve (AUC) is calculated. In addition, we compared CMI2NI with several PC algorithm-based methods, such as pcalg (49) and PCA-CMI (28).

## RESULTS

### Simulation study

For simulation expression data, the widely used benchmark networks along with expression datasets from DREAM challenge were adopted here to evaluate our method. The gold standard networks were selected from source networks of real species. The expression data were generated with the nonlinear ordinary differential equation (ODE) systems in which the network structures were determined with detailed dynamics of both transcriptional and translational processes (50). In this work, the DREAM3 datasets about *Yeast* knock-out gene expression data with sizes 10, 50 and 100 were used (4).

**Figure 3.**
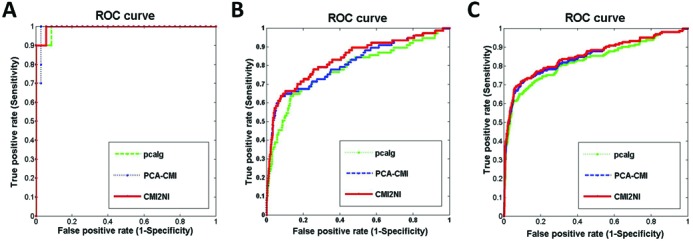
ROC curves of several methods on *Yeast* networks of sizes 10, 50 and 100 from DREAM3 challenge. The red solid line denotes the ROC curve by CMI2NI, while the green and blue ones respectively denote the ROC curves by PCA-CMI and pcalg. (**A**) The ROC curves on networks of size 10. (**B**) The ROC curves on networks of size 50. (**C**) The ROC curves on networks of size 100.

Firstly, we tested CMI2NI on the Yeast gene expression data with network size 10 and sample number 10. We chose 0.03 as the threshold value of mutual information and conditional mutual inclusive information to decide independence, and the order index was not constrained, i.e. the algorithm terminated until there was no more higher order CMI2 to be computed. In order to evaluate the performance of CMI2NI, the AUC score was adopted. As shown in Figure 3A, CMI2NI performs best with an AUC score of 0.994, implying the efficiency of CMI2NI. In addition, we also compared CMI2NI with partial correlation coefficient-based PC algorithm (pcalg) (49) and conditional mutual information-based PC-algorithm (PCA-CMI) (28). The results can be found in Figure 3A, where we can see that CMI2NI is superior to the other methods. The detailed results of different approaches can be found in Table 1 with respect to PPV, ACC, MCC and AUC. From Table 1, we can see that CMI2NI and PCA-CMI perform comparatively well with respect to PPV, ACC and MCC, and both approaches outperform pcalg.

**Table 1.** Comparison of different methods on networks with sizes 10, 50 and 100 in DREAM3 challenge

Method	TP	FP	TN	FN	TPR	FPR	PPV	ACC	MCC	AUC
**Size 10**										
pcalg	20	8	62	0	1.000	0.114	0.714	0.911	0.795	0.991
PCA-CMI	18	2	68	2	0.900	0.028	0.900	0.956	0.871	0.991
CMI2NI	18	2	68	2	0.900	0.028	**0.900**	**0.956**	**0.871**	**0.994**
**Size 50**										
pcalg	66	162	2134	88	0.428	0.071	0.289	0.898	0.299	0.782
PCA-CMI	72	78	2218	82	0.467	0.034	0.480	0.934	0.438	0.810
CMI2NI	78	80	2216	76	0.506	0.035	**0.493**	**0.936**	**0.466**	**0.834**
**Size 100**										
pcalg	152	252	9316	180	0.457	0.026	0.376	0.956	0.392	0.829
PCA-CMI	114	96	9472	218	0.343	0.010	0.542	0.968	0.416	0.849
CMI2NI	128	76	9492	204	0.385	0.007	**0.627**	**0.971**	**0.478**	**0.855**

Secondly, we tested CMI2NI on the Yeast gene expression data with network size 50 and sample number 50. The network containing 50 nodes with 77 edges was selected from real and experimental verified networks. We set the threshold value 0.05 for MI and CMI2 to decide independence and the order index was not constrained. As shown in Figure 3B, CMI2NI outperforms other reference methods with the highest AUC score of 0.834. From Table 1, we can see that CMI2NI performs better than the other methods with respect to all the metrics listed. For example, CMI2NI achieved 0.492, 0.396, 0.397 and 0.834 for PPV, ACC, MCC and AUC, respectively.

Thirdly, we tested CMI2NI on the *Yeast* gene expression data with network size 100 and sample number 100. The reference network contains 100 nodes with 166 edges. We set the threshold value 0.03 of MI and CMI2 to decide independence and the order index was set to the first order which is a usually adopted order for large-scale networks to reduce the computational burden. As shown in Figure 3C, CMI2NI performs better than other reference methods with the highest AUC value of 0.856. The detailed results of different approaches can be found in Table 1 with respect to various metrics. From Table 1, we can see that CMI2NI performs better than the other two methods with the highest values 0.628, 0.972, 0.479 and 0.856 for PPV, ACC, MCC and AUC, respectively. In the 100-gene network from DREAM dataset, 14 edges were detected by CMI2 but missed by CMI. Furthermore, CMI2 successfully silenced 20 edges overestimated by CMI without reducing the true positive rate in the 100-gene network.

The results on all the three datasets with different network sizes from DREAM challenge demonstrated the effectiveness of our CMI2NI. Furthermore, the good performance of CMI2NI indicates that CMI2, as a new measure of causal regulation strength, is superior to CMI.

### Reconstruction of SOS network in *Escherichia coli*

Besides the above simulation datasets, CMI2NI was also applied to reconstruct gene networks from real gene expression data. We evaluated our CMI2NI on the well-known SOS DNA repair network which is an experimentally verified network in *E. coli* with real gene expression data (47,48).

The network is a 9-gene sub-network of SOS pathway in *E. coli*. The SOS pathway, which regulates cell survival and repair after DNA damage, involves the *lexA* and *recA* genes. There are more than 30 genes that are directly regulated by *lexA* and *recA*, while tens or even hundreds of other genes that are indirectly regulated by the two genes. Here, the nine transcripts in the test network include the principle mediators of the SOS response (*lexA* and *recA*), four other regulatory genes with known involvement in the SOS response (*ssb, recF, dinI* and *umuDC*), and three sigma factor genes (*rpoD, rpoH* and *rpoS*) whose regulations play important roles in the SOS response. For the expression data, we chose the perturbation data, which were obtained after perturbations were applied to the test network in *E. coli*. In the perturbation, each of the nine genes in the test network is overexpressed with arabinose-controlled episomal expression plasmid, and the change in expression of each transcript relative to the unperturbed cells was accordingly measured using quantitative real-time polymerase chain reaction (qPCR).

**Figure 4.**
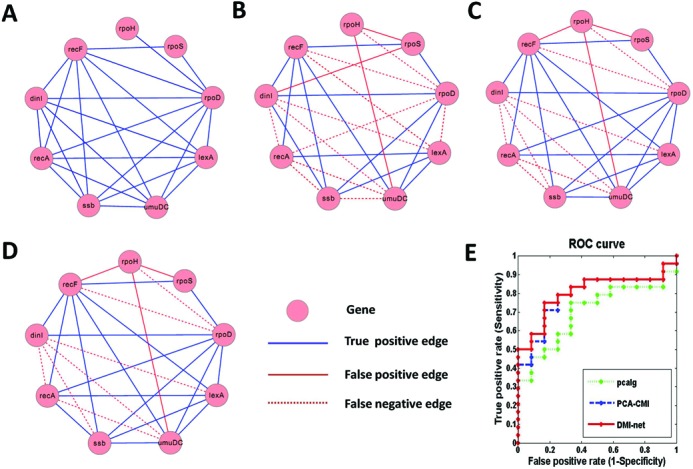
Comparison of CMI2NI with pcalg and PCA-CMI on the SOS network. (**A**) Benchmark (true) network. (**B**) Network inferred by pcalg. (**C**) Network inferred by PCS-CMI. (**D**) Network inferred by CMI2NI. In the network, a pink node represents a gene. (**E**) ROC curves of three methods, where the AUC score of CMI2NI is 0.802.

The performance of CMI2NI was evaluated with the network of size 9 and the expression dataset generated under perturbations. We chose 0.01 as the threshold of both MI and CMI2. To show the performances of CMI2NI and other methods, the true network and inferred networks were visualized with Cytoscape (51). Figure 4A shows the true network with 24 edges. Figure 4B–D shows the networks inferred by pcalg, PCA-CMI and CMI2NI, respectively, with corresponding ACC of 0.583, 0.694 and 0.722. From the results shown in Figure 4E, we can see that CMI2NI outperforms the other two methods significantly, where CMI2NI achieves the highest AUC of 0.802.

The detailed results of different methods can be found in Table 2 with respect to some indexes, such as PPV, ACC, MCC, etc. From Table 2, we can see that CMI2NI performs best on most metrics. Although pcalg achieves higher TPR with the same parameter, the higher FPR disables it as a good performer. Both PCA-CMI and CMI2NI outperform pcalg, implying the efficiency of CMI. The superior performance of CMI2NI over PCA-CMI demonstrates that CMI2 is better than CMI when quantifying the causal strength between variables.

**Table 2.** Comparison of different methods on SOS DNA repair network

Method	TP	FP	TN	FN	TPR	FPR	PPV	MCC	ACC	AUC
pcalg	12	3	9	12	0.500	0.250	0.800	0.239	0.583	0.701
PCA-CMI	16	3	9	8	0.667	0.250	0.842	0.393	0.694	0.792
CMI2NI	17	3	9	7	0.708	0.250	**0.850**	**0.435**	**0.722**	**0.802**

### Performance of CMI2NI

The theoretical analysis of CMI2 has proven that CMI2 can be decomposed into CMI and a non-negative term. This implies that CMI2 value is bigger than CMI. Hence, CMI2 can address the underestimation problem of CMI. To further test this theoretical conclusion, we investigated the value of CMI2 with both simulation and real expression datasets. Moreover, we compared values of CMI2 and CMI. For simulation dataset, we used a random dataset as well as the dataset from DREAM challenge. For real gene expression dataset, we used the expression data of SOS network in *E. coli*.

**Figure 5.**
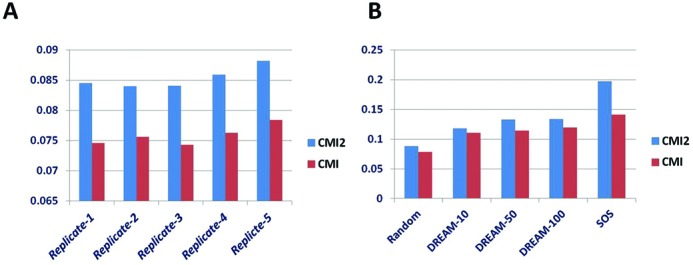
Comparison of methods CMI2 and CMI in quantifying causal strengths in different datasets. Blue and red bars represent CMI2 and CMI respectively. (**A**) Comparison on five replicate random datasets. (**B**) Comparison on random, DREAM and SOS datasets.

Firstly, we generated random variables with different samples 5, 10, 100 and 500, we took one or more of them as conditional variable(s). We computed their CMI2 and CMI values using these datasets. The calculation was performed 100 times on each dataset, and the mean of the CMI2 and CMI values were used for comparison. Figure 5A gives the results of CMI2 and CMI values. From the histogram, we can find that for all the types of datasets with different samples, CMI2 values are always larger than CMI values, which is consistent with theoretical analysis of CMI2.

Secondly, we investigated the values of CMI2 on the datasets from DREAM challenge and the real expression dataset for SOS network in *E. coli*. When computing the causal strength, we only calculated CMI2 or CMI for the edges in the network, where the means of all edges for CMI2s or CMIs were used for comparison. Figure 5B shows the values of CMI2 and CMI on the five datasets, from which we can clearly see that CMI2 is indeed higher than CMI.

### Case study: reconstruction of cancer-specific gene regulatory network

It is well recognized that most complex traits are caused by the dysfunction of certain functional modules and pathways (52), where the gene regulatory circuit may be rewired in the diseases. Since the gene regulatory network provides a global view of the gene regulations, we hereby investigated how the cancer genes are regulated by constructing a gene regulatory network for cancer. As a case study, we applied CMI2NI to build a GRN for acute myeloid leukemia (AML) based on the RNA sequencing data of a large cohort of AML patients from TCGA (http://cancergenome.nih.gov/) (53,54). The Level-3 processed data was used here, and the RPKM (read per kilobase of exon per million mapped reads) value was used as the gene expression value.

**Figure 6.**
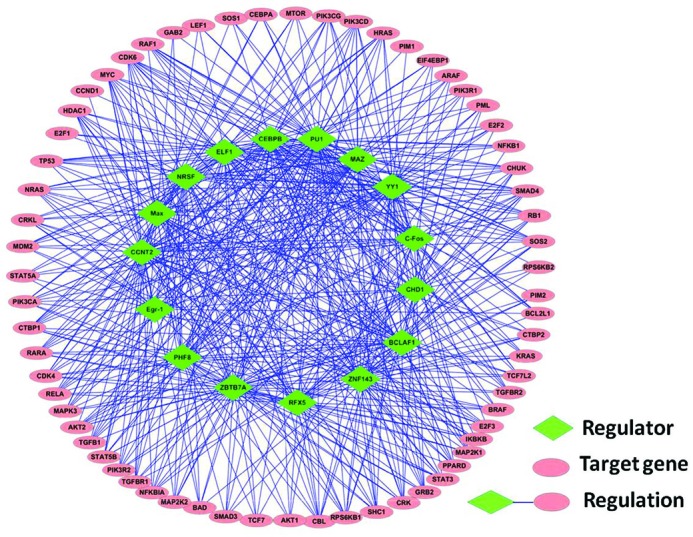
AML-specific gene regulatory network reconstructed by CMI2NI.

Figure 6 shows the AML-specific GRN constructed by CMI2NI. In particular, we considered the 81 cancer genes involved in a network built by RACER (55), where the network was constructed based on the same AML gene expression dataset used here. In the AML-specific GRN, there are 16 regulators and 65 target genes, and we inferred the regulation relationships between the regulators and genes with CMI2NI. In total, we detected 550 regulations, among which 113 regulations have been reported by RACER. Compared with the network inferred by RACER, there are two hub regulators NRSF and BCLAF1 in the GRN constructed by CMI2NI, where these two regulators respectively target 39 and 35 genes. In the GRN built by RACER, the degrees of these two regulators are small. By investigating the target genes of NRSF and BCLAF1 from our constructed GRN, we want to see which pathways these two regulators are involved in and how they are related to cancer. With the cancer gene annotation system CaGe (http://mgrc.kribb.re.kr/cage/), we noticed that the genes targeted by NRSF are significantly enriched in cancer pathways and the top two ranked pathways are leukemia-specific pathways (detailed results can be found in Supplementary Tables S1). Similarly, BCLAF1 target genes are also significantly enriched in leukemia-specific pathways (detailed results can be found in Supplementary Tables S2). Despite these two regulators have not been reported directly to be related to leukemia, the analysis of their target genes implies that they play important roles in leukemia. In the future, these two regulators can be considered when designing target therapy for leukemia.

## DISCUSSION

The information theory-based association measures, e.g. MI and CMI have been widely used to infer gene regulation networks. However, these measurements either underestimate or overestimate the associations between variables (5,56). In this paper, we proposed a novel association concept, namely CMI2, to accurately quantify the dependency between a pair of variables. CMI2 provides a natural generalization of correlation and is capable of characterizing the nonlinear dependency between variables that is common in biology. Furthermore, CMI2NI can accurately quantify the causal strengths or correlations between gene-pairs so that the indirect regulation can be eliminated, which is the key point to improve the accuracy of GRN inference. With CMI2, we developed a network inference algorithm, namely CMI2NI, to infer gene regulation networks. With the power of PC algorithm, CMI2NI can also keep the natural sparseness of biological networks. The benchmark results show that CMI2NI outperforms other popular approaches, implying the effectiveness of CMI2NI. Considering the complex functional relationships among genes (57), we also constructed an AML-specific GRN with CMI2NI to see how the cancers genes are regulated. Similar to other network-based approaches, such as network differentiation (58–63) and dynamical network biomarkers (64–66), investigating the regulatory circuit of cancer genes provides new insights into the cancer pathogenesis.

Despite the advantages of CMI2NI, we notice there is still room to improve it. Firstly, similar to PCA-CMI, CMI2NI cannot directly infer edge directionality, which is also a general problem of many other methods, especially for those not working on time series data (23). Secondly, it is still a challenge task to select the conditional genes in an optimization way. In the PC-algorithm, genes *i, j* and their neighbours are randomly selected when calculating association. For example, there are *T* (*T* $ \ge$ 1) genes which are adjacent with both genes *i* and *j*. When constructing the *L*th-order (*L* $ \le$ *T*) network, all the *L*th-order CMI2s for the possible combinations of *L* conditional genes from *T* genes are computed, and the maximal one or the geometric mean of them is used to describe the regulation strength. However, the selection of conditional genes may affect the performance of PC algorithm.

## AVAILABILITY

The software CMI2NI is freely accessible at http://www.comp-sysbio.org/cmi2ni.

## SUPPLEMENTARY DATA

Supplementary Data are available at NAR Online.

SUPPLEMENTARY DATA
