# Supplementary material for: Conditional mutual inclusive information enables accurate quantification of associations in gene regulatory networks
Source: Nucleic Acids Res. 2014 Dec 24;43(5):e31. doi: 10.1093/nar/gku1315 (PMC4357691; doi:10.1093/nar/gku1315)
Supplement: SUPPLEMENTARY DATA [file supp_43_5_e31__index.html]

Conditional mutual inclusive information enables accurate quantification of associations in gene regulatory networks — SUPPLEMENTARY DATA 

# Conditional mutual inclusive information enables accurate quantification of associations in gene regulatory networks

## SUPPLEMENTARY DATA

**Files in this Data Supplement:**

- SUPPLEMENTARY DATA
