# Supplementary material for: Conditional mutual inclusive information enables accurate quantification of associations in gene regulatory networks
Source: Nucleic Acids Res. 2014 Dec 24;43(5):e31. doi: 10.1093/nar/gku1315 (PMC4357691; doi:10.1093/nar/gku1315)
Supplement: SUPPLEMENTARY DATA [file supp_gku1315_nar-02648-met-n-2014-File002.pdf]

Supplementary Data

Conditional mutual inclusive information enables accurate quantification of associations in gene regulatory networks

Xiujun Zhang[1,2,3], Juan Zhao[1], Jin-Kao Hao[4], Xing-Ming Zhao[5,]* and Luonan Chen[1,]*

[1] Key Laboratory of Systems Biology, Shanghai Institutes for Biological Sciences, Chinese Academy of Sciences, Shanghai 200031, China

[2] Department of Mathematics, Xinyang Normal University, Xinyang 464000, China

[3] School of Chemical and Biomedical Engineering, Nanyang Technological University, Singapore 637459, Singapore

[4] LERIA, Department of Computer Science, University of Angers, Angers 49045, France

[5] Department of Computer Science, School of Electronics and Information Engineering, Tongji University, Shanghai 201804, China

* To whom correspondence should be addressed to Luonan Chen. Tel: +86 21 6436 5937; Fax:+86 21 5492 0120; Email: lnchen@sibs.ac.cn. Correspondence may also be addressed to Xing-Ming Zhao. Tel: +86 21 6958 3959; Fax: +86 21 6958 3959; Email: xm_zhao@tongji.edu.cn.

1. Associations quantification and mutual information

Association or interaction between two genes in a gene regulatory network (GRN) is strength of causality or regulation between genes. Accurate measure of associations plays an important role in GRN inference. Correlation-based methods have been widely used to measure associations in GRN inference and achieved great success, such as Pearson correlation coefficient (PCC) and mutual information (MI) (1,2).

Recently, mutual information (MI) has been widely used to reconstruct GRNs for its advantage in measuring the dependence of variables. Although MI performs excellent in measuring association in a simple network with only two nodes (variables), it has been proved over-estimating for its blindness to the strength of indirect associations in a complex network with triple or more nodes. To address this issue, conditional mutual information (CMI) was proposed to tune the over-estimation and has been regarded as a bright amendment to MI (3). As an extension of MI, CMI has been successfully used to construct GRNs combining with MI (4). Moreover, CMI is also widely used to discover some other regulatory modes in systems biology and achieved its

successfulness. However, the theoretical analysis proves that CMI is also imperfect for its under-estimation in some situations and the quantification of causal strength is still a non-trivial question. So how to accurately quantify association between two genes (variables) is still a challenging task in GRN inference(5-7).

As is well known, a GRN is complicated in topology which results in the biological complexity. Any complex network is composed of small units such as two-node and triple-node networks. Accurately measuring association between genes in these small units is the key point to improve efficiency of network inference. Most popular methods such as Pearson correlation coefficient and mutual information-based methods only address two-edge networks. The performances of these methods are not satisfactory enough because they cannot exclude the indirect associations from third one(s). Recently some new methods addressing triple-edge networks such as partial correlation coefficient and conditional mutual information-based methods achieved certain success in network inference. In particular, a new association measure is proposed based on the relative entropy distance (6-7). In this work, we will study how to accurately measure the associations between genes in complex structures of GRNs.

2. KL-divergence-based casual strength measure

Recently, to accurately measure the causal strength between two genes, a measure based on Kullback–Leibler (KL) divergence was proposed(6). Fig.S1(**B**) gives a DAG with three variables *X*, *Y* and *Z*. Strength of a causal arrow measures causal strength of one arrow between two variables in a directed acyclic graph (DAG) (6). In this DAG, variable *Y* is regulated by variable *Z* both directly and mediated by variable *X*. The causal strength of arrow $X \to Y$ is defined as

$$\mathcal{C}_{X\to Y}(X;Y|Z) = D_{KL}(P(X,Y,Z)\,||\,P_{X\to Y}(X,Y,Z)), \tag{S1}$$

where $P(X,Y,Z)$ is the joint probability of *X*, *Y* and *Z*, $P_{X\to Y}(X,Y,Z)$ is the joint probability of *X*, *Y* and *Z* for removing arrow $X\to Y$, and $D_{KL}(P\,||\,P_{X\to Y})$ is Kullback–Leibler (K-L) divergence from $P(X,Y,Z)$ to $P_{X\to Y}(X,Y,Z)$ (6-7).

3. Conditional mutual inclusive information (CMI2)

In a directed acyclic graph (DAG), the association between *X* and *Y* given Z can be divided into two parts, i.e. $X \rightarrow Y$ and $Y \rightarrow X$. Conditional mutual inclusive information (CMI2) between *X* and *Y* given *Z* is defined as

$$CMI2(X,Y \mid Z) = \left(D_{KL}(P \,\|\, P_{X\rightarrow Y}) + D_{KL}(P \,\|\, P_{Y\rightarrow X})\right)/2, \qquad \text{(S2)}$$

where $P = P(X,Y,Z)$ is the joint probability distribution of *X*, *Y* and *Z*, $P_{X\rightarrow Y} = P_{X\rightarrow Y}(X,Y,Z)$ and $P_{Y\rightarrow X} = P_{Y\rightarrow X}(X,Y,Z)$ are the interventional probability distributions of *X*, *Y* and *Z* for removing edge $X \rightarrow Y$ and $Y \rightarrow X$, respectively. $D_{KL}(P \,\|\, P_{X\rightarrow Y})$ and $D_{KL}(P \,\|\, P_{Y\rightarrow X})$ are KL-divergences from P to $P_{X\rightarrow Y}$ and $P_{Y\rightarrow X}$. Similar to CMI, CMI2 has an order number |*Z*|, i.e. the number of conditional variables *Z*, and MI can be regarded as zero-order CMI2.

The probability $P_{X\rightarrow Y}$ is defined (6) as

$$P_{X\rightarrow Y}(x,y,z) = P(x,z)\sum_{x} P(y \mid z,x)P(x), \qquad \text{(S3)}$$

where $P(y \mid z,x)$ is conditional probability. According to the definition of K-L divergence (7,8), $D_{KL}(P \,\|\, P_{X\rightarrow Y})$ (same for $D_{KL}(P \,\|\, P_{Y\rightarrow X})$) can be rewritten as

$$D_{KL}(P \,\|\, P_{X\rightarrow Y}) = \sum_{x,y,z} P(x,y,z)\ln\frac{P(x,y,z)}{P(x,z)\sum_{x} P(y \mid z,x)P(x)}. \qquad \text{(S4)}$$

The above quantity is proved to be a decomposition including conditional mutual information (CMI).

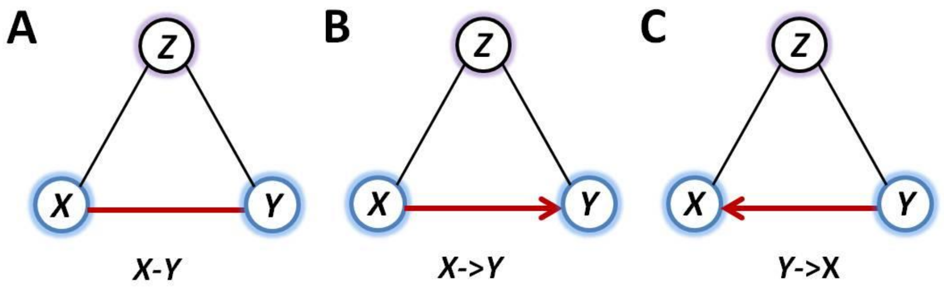

Fig.S1. The association between two variables (genes) *X* and *Y* in a triple-node network (**A**). The association can be divided into two parts, i.e. *X->Y* (**B**) and *Y->X* (**C**) .

The decomposition can be described as follows.

With the probability theory, the conditional mutual inclusive information (CMI2) can be decomposed into

$$CMI2(X;Y|Z) = CMI(X;Y|Z) + \tfrac{1}{2} D_{KL}(P(Y|Z) \| P_{X\to Y}(Y|Z)) + \tfrac{1}{2} D_{KL}(P(X|Z) \| P_{Y\to X}(X|Z)). \quad \text{(S5)}$$

where $CMI(X;Y|Z)$ is conditional mutual information, $D_{KL}(P(Y|Z) \| P_{X\to Y}(Y|Z))$ is KL-divergence between two conditional distributions.

Proof. Due to $P_{X\to Y}(x,y,z) = P(x,z)\sum_x P(y|z,x)P(x)$, we have

$$\begin{aligned}
D_{KL}(P(X,Y,Z) \| P_{X\to Y}(X,Y,Z)) &= \sum_{x,y,z} P(x,y,z)\log\frac{P(x,y,z)}{P_{X\to Y}(x,y,z)} \\
&= \sum_{x,y,z} P(x,y,z)\log\frac{P(y|x,z)P(x,z)}{P(x,z)\sum_x P(y|z,x)P(x)} \\
&= \sum_{x,y,z} P(x,y,z)\log\frac{P(y|x,z)}{\sum_x P(y|z,x)P(x)} \\
&= \sum_{x,y,z} P(x,y,z)\log\frac{P(y|x,z)}{P(y|z)} + \sum_{x,y,z} P(x,y,z)\log\frac{P(y|z)}{\sum_x P(y|z,x)P(x)} \\
&= \sum_{x,y,z} P(x,y,z)\log\frac{P(x,y|z)P(z) \big/ P(x|z)P(z)}{P(y|z)} + \sum_{x,y,z} P(x,y,z)\log\frac{P(y|z)}{\sum_x P(y|z,x)P(x)} \\
&= \sum_{x,y,z} P(x,y,z)\log\frac{P(x,y|z)}{P(x|z)P(y|z)} + \sum_{x,y,z} P(x,y,z)\log\frac{P(y|z)}{\sum_x P(y|z,x)P(x)} \\
&= I(X;Y|Z) + D_{KL}(P(Y|Z) \| P_{X\to Y}(Y|Z))
\end{aligned}$$

The second term will vanish if variables *X* and *Z* are independent which can be inferred by $P_{X\to Y}(Y|Z) = P(Y|Z)$ because $P_{X\to Y}(y|z) = \sum_x P(y|x,z)P(x) = \sum_x P(y|x,z)P(x|z) = P(y|z)$.

Similarly, $D_{KL}(P(X,Y,Z) \| P_{Y\to X}(X,Y,Z)) = I(X;Y|Z) + D_{KL}(P(X|Z) \| P_{Y\to X}(X|Z))$. With above equations, we have the result in Eq(S5)

With above decomposition, the traits on association measures can be remarked as follows. The expression level of gene Z is equal to X (or Y) will result in $CMI(X;Y|Z)=0$ which is the reason for CMI's under-estimation of associations. When the expression level of gene Z is near or equal to X (or Y), which can be measured with Pearson correlation coefficient near or equal to 1, the joint entropy formula have the following equals.

H(Z)=H(X,Z)=H(X), H(Y,Z)=H(X,Y,Z)=H(X,Y).

So the conditional mutual information CMI(X,Y|Z) will be equal to zero, i.e.

CMI(X,Y|Z)=H(X,Z)+H(Y,Z)-H(X,Y,Z)-H(Z)=H(X)+H(X,Y)-H(X,Y)-H(X) =0.

With additional non-negative item $D_{KL}(P(Y|Z)\|P_{X\to Y}(Y|Z))$, $D_{KL}(P\|P_{X\to Y})$ partially addresses the under-estimation because Z=X will not result in $D_{KL}(P(Y|Z)\|P_{X\to Y}(Y|Z))=0$ but Z=Y will result in $D_{KL}(P(Y|Z)\|P_{X\to Y}(Y|Z))=0$.

4. Computation of conditional mutual inclusive information (CMI2)

In order to estimate conditional mutual inclusive information(CMI2), we give the hypothesis that the variables have a multivariate Gaussian distribution which has been widely proved to be reasonable.

Theorem 1 Let *X* and *Y* be 1-dimension variables, *Z* a $n_z(n_z \ge 1)$ -dimension variable, and *X*, *Y* and *Z* follow Gaussian distribution. Then

$$CMI2(X;Y|Z)=\frac{1}{4}\left(tr(C^{-1}\Sigma)+tr(\tilde{C}^{-1}\tilde{\Sigma})+\ln C_0+\ln\tilde{C}_0-2n\right), \qquad (10)$$

where

$$n=n_z+2,\ C_0=\rho_{xx}\left(\left(\Sigma^{-1}\right)_{xx}-\left(\Sigma_1^{-1}\right)_{xx}+\rho_{xx}{}^{-1}\right),\ \tilde{C}_0=\rho_{yy}\left(\left(\tilde{\Sigma}^{-1}\right)_{yy}-\left(\tilde{\Sigma}_1^{-1}\right)_{yy}+\rho_{yy}{}^{-1}\right),$$

$$C=\begin{pmatrix} C_{xx} & C_{xy} & C_{xz} \\ C_{xy} & C_{yy} & C_{yz} \\ C_{xz}{}^T & C_{yz}{}^T & C_{zz}\end{pmatrix}^{-1},\ \tilde{C}=\begin{pmatrix} \tilde{C}_{yy} & \tilde{C}_{yx} & \tilde{C}_{yz} \\ \tilde{C}_{yx} & \tilde{C}_{xx} & \tilde{C}_{xz} \\ \tilde{C}_{yz}{}^T & \tilde{C}_{xz}{}^T & \tilde{C}_{zz}\end{pmatrix}^{-1},$$

$$C_{xx}=\left(\Sigma_1^{-1}\right)_{xx}, C_{xy}=0, C_{xz}=\left(\Sigma_1^{-1}\right)_{xz},$$
$$C_{yy}=\left(\Sigma^{-1}\right)_{yy}-\left(\Sigma^{-1}\right)^2_{xy}\left(\left(\Sigma^{-1}\right)_{xx}-\left(\Sigma_1^{-1}\right)_{xx}+\rho_{xx}{}^{-1}\right)^{-1},$$
$$C_{yz}=\left(\Sigma^{-1}\right)_{yz}-\left(\Sigma^{-1}\right)_{xy}\left(\left(\Sigma^{-1}\right)_{xx}-\left(\Sigma_1^{-1}\right)_{xx}+\rho_{xx}{}^{-1}\right)^{-1}\left(\left(\Sigma^{-1}\right)_{xz}-\left(\Sigma_1^{-1}\right)_{xz}\right),$$
$$C_{zz}=\left(\Sigma^{-1}\right)_{zz}-\left(\left(\Sigma^{-1}\right)_{xx}-\left(\Sigma_1^{-1}\right)_{xx}+\rho_{xx}{}^{-1}\right)^{-1}\left(\left(\Sigma^{-1}\right)_{xz}{}^T-\left(\Sigma_1^{-1}\right)_{xz}{}^T\right)\left(\left(\Sigma^{-1}\right)_{xz}-\left(\Sigma_1^{-1}\right)_{xz}\right),$$

$$\tilde{C}_{yy}=\left(\tilde{\Sigma}_1^{-1}\right)_{yy}, \tilde{C}_{yx}=0, \tilde{C}_{yz}=\left(\tilde{\Sigma}_1^{-1}\right)_{yz},$$
$$\tilde{C}_{xx}=\left(\tilde{\Sigma}^{-1}\right)_{xx}-\left(\tilde{\Sigma}^{-1}\right)^2_{yx}\left(\left(\tilde{\Sigma}^{-1}\right)_{yy}-\left(\tilde{\Sigma}_1^{-1}\right)_{yy}+\rho_{yy}{}^{-1}\right)^{-1},$$
$$\tilde{C}_{xz}=\left(\tilde{\Sigma}^{-1}\right)_{xz}-\left(\tilde{\Sigma}^{-1}\right)_{yx}\left(\left(\tilde{\Sigma}^{-1}\right)_{yy}-\left(\tilde{\Sigma}_1^{-1}\right)_{yy}+\rho_{yy}{}^{-1}\right)^{-1}\left(\left(\tilde{\Sigma}^{-1}\right)_{yz}-\left(\tilde{\Sigma}_1^{-1}\right)_{yz}\right),$$
$$\tilde{C}_{zz}=\left(\tilde{\Sigma}^{-1}\right)_{zz}-\left(\left(\tilde{\Sigma}^{-1}\right)_{yy}-\left(\tilde{\Sigma}_1^{-1}\right)_{yy}+\rho_{yy}{}^{-1}\right)^{-1}\left(\left(\tilde{\Sigma}^{-1}\right)_{yz}{}^T-\left(\tilde{\Sigma}_1^{-1}\right)_{yz}{}^T\right)\left(\left(\tilde{\Sigma}^{-1}\right)_{yz}-\left(\tilde{\Sigma}_1^{-1}\right)_{yz}\right),$$

$$\Sigma_1=\begin{pmatrix}\rho_{xx} & \rho_{xz}\\ \rho_{xz} & \rho_{zz}\end{pmatrix},\ \tilde{\Sigma}_1=\begin{pmatrix}\rho_{yy} & \rho_{yz}\\ \rho_{yz} & \rho_{zz}\end{pmatrix},\ \Sigma=\begin{pmatrix}\rho_{xx} & \rho_{xy} & \rho_{xz}\\ \rho_{xy} & \rho_{yy} & \rho_{yz}\\ \rho_{xz} & \rho_{yz} & \rho_{zz}\end{pmatrix},\ \tilde{\Sigma}=\begin{pmatrix}\rho_{yy} & \rho_{yx} & \rho_{yz}\\ \rho_{yx} & \rho_{xx} & \rho_{xz}\\ \rho_{yz} & \rho_{xz} & \rho_{zz}\end{pmatrix}.$$

Proof. The probability density function of X is

$$P(x)=(2\pi)^{-\frac{1}{2}}\rho_{xx}{}^{-\frac{1}{2}}\exp\left(-\frac{1}{2}(x-\mu_x)\rho_{xx}{}^{-1}(x-\mu_x)\right), \tag{S7}$$

where μ_x is the mean value of x and ρ_{xx} is correlation of x.

Let $u=(x,y,z)^T$ and $v=(x,z)^T$, where z is a $n_z(\geq 1)$ -dimension variable, then the joint probability

$$\begin{aligned}P(x,y,z)&=(2\pi)^{-\frac{n}{2}}(\det\Sigma)^{-\frac{1}{2}}\exp\left(-\frac{1}{2}(u-\mu_u)^T\Sigma^{-1}(u-\mu_u)\right)\\ &=(2\pi)^{-\frac{n}{2}}(\det\Sigma)^{-\frac{1}{2}}\exp\{-\frac{1}{2}(x-\mu_x)\left(\Sigma^{-1}\right)_{xx}(x-\mu_x)-\frac{1}{2}(y-\mu_y)\left(\Sigma^{-1}\right)_{yy}(y-\mu_y)\\ &-\frac{1}{2}(z-\mu_z)^T\left(\Sigma^{-1}\right)_{zz}(z-\mu_z)-(x-\mu_x)\left(\Sigma^{-1}\right)_{xy}(y-\mu_y)-(x-\mu_x)\left(\Sigma^{-1}\right)_{xz}(z-\mu_z)\\ &-(y-\mu_y)\left(\Sigma^{-1}\right)_{yz}(z-\mu_z)\},\end{aligned} \tag{S8}$$

$$\begin{aligned}P(x,z)&=(2\pi)^{-\frac{n-1}{2}}(\det\Sigma_1)^{-\frac{1}{2}}\exp\left(-\frac{1}{2}(v-\mu_v)^T\Sigma_1^{-1}(v-\mu_v)\right)\\ &=(2\pi)^{-\frac{n-1}{2}}(\det\Sigma_1)^{-\frac{1}{2}}\exp\{-\frac{1}{2}(x-\mu_x)\left(\Sigma_1^{-1}\right)_{xx}(x-\mu_x)-(x-\mu_x)\left(\Sigma_1^{-1}\right)_{xz}(z-\mu_z)\\ &-\frac{1}{2}(z-\mu_z)^T\left(\Sigma_1^{-1}\right)_{zz}(z-\mu_z)\},\end{aligned} \tag{S9}$$

where $n=n_z+2$, μ_u is the mean value of u, μ_v is the mean value of v, Σ is the covariance matrix of x, y and z, Σ_1 is the covariance matrix of x and z, i.e.

$$\Sigma=\begin{pmatrix}\rho_{xx} & \rho_{xy} & \rho_{xz}\\ \rho_{xy} & \rho_{yy} & \rho_{yz}\\ \rho_{xz} & \rho_{yz} & \rho_{zz}\end{pmatrix},\Sigma_1=\begin{pmatrix}\rho_{xx} & \rho_{xz}\\ \rho_{xz} & \rho_{zz}\end{pmatrix},$$ $(\Sigma)_{ij}, i=x,y,z; j=x,y,z$ is the $i\times j$ item of the matrix Σ.

So with Eqs(S7), (S8) and (S9), we have

$$\begin{aligned}P(y\,|\,z,x)P(x)&=\frac{P(x,y,z)}{P(x,z)}P(x)\\ &=(2\pi)^{-1}\left(\rho_{xx}\frac{\det\Sigma}{\det\Sigma_1}\right)^{-\frac{1}{2}}\exp\left\{-\frac{1}{2}(x-\mu_x)\left[\left(\Sigma^{-1}\right)_{xx}-\left(\Sigma_1^{-1}\right)_{xx}+\rho_{xx}{}^{-1}\right](x-\mu_x)\right.\\ &-(x-\mu_x)\left[\left(\Sigma^{-1}\right)_{xy}(y-\mu_y)+\left(\left(\Sigma^{-1}\right)_{xz}-\left(\Sigma_1^{-1}\right)_{xz}\right)(z-\mu_z)\right]\\ &\left.-\frac{1}{2}(y-\mu_y)\left(\Sigma^{-1}\right)_{yy}(y-\mu_y)-(y-\mu_y)\left(\Sigma^{-1}\right)_{yz}(z-\mu_z)-\frac{1}{2}(z-\mu_z)^T\left[\left(\Sigma^{-1}\right)_{zz}-\left(\Sigma_1^{-1}\right)_{zz}\right](z-\mu_z)\right\}.\end{aligned} \tag{S10}$$

If A is a symmetric positive-definite matrix, then

$$\sum_x e^{-\frac{1}{2}\sum_{i,j=1}^{n} A_{ij}x_i x_j + \sum_{i=1}^{n} B_i x_i} = (2\pi)^{\frac{n}{2}} (\det A)^{-\frac{1}{2}} e^{\frac{1}{2}B^T A^{-1} B}, \qquad \text{(S11)}$$

For $\left(\Sigma^{-1}\right)_{xx} - \left(\Sigma_1^{-1}\right)_{xx} + \rho_{xx}{}^{-1}$ is a scalar, so with Eqs (S10) and (S11), we have

$$\begin{aligned}
\sum_x P(y \mid z, x)P(x) &= (2\pi)^{-1} \left(\rho_{xx} \frac{\det \Sigma}{\det \Sigma_1} \right)^{-\frac{1}{2}} (2\pi)^{\frac{1}{2}} \left(\left(\Sigma^{-1}\right)_{xx} - \left(\Sigma_1^{-1}\right)_{xx} + \rho_{xx}{}^{-1} \right)^{-\frac{1}{2}} \cdot \\
& \exp\left\{ \frac{1}{2} \left[\left(\Sigma^{-1}\right)_{xy} (y-\mu_y) + \left(\left(\Sigma^{-1}\right)_{xz} - \left(\Sigma_1^{-1}\right)_{xz} \right)(z-\mu_z) \right] \left[\left(\Sigma^{-1}\right)_{xx} - \left(\Sigma_1^{-1}\right)_{xx} + \rho_{xx}{}^{-1} \right]^{-1} \right. \\
& \quad \left. \left[\left(\Sigma^{-1}\right)_{xy} (y-\mu_y) + \left(\left(\Sigma^{-1}\right)_{xz} - \left(\Sigma_1^{-1}\right)_{xz} \right)(z-\mu_z) \right] \right\} \cdot \\
& \exp\left\{ -\frac{1}{2}(y-\mu_y)\left(\Sigma^{-1}\right)_{yy}(y-\mu_y) - (y-\mu_y)\left(\Sigma^{-1}\right)_{yz}(z-\mu_z) - \frac{1}{2}(z-\mu_z)^T \left[\left(\Sigma^{-1}\right)_{zz} - \left(\Sigma_1^{-1}\right)_{zz} \right] (z-\mu_z) \right\}.
\end{aligned} \qquad \text{(S12)}$$

With Eqs (S9), (S12) and $\left(\Sigma^{-1}\right)_{xz}(z-\mu_z) = (z-\mu_z)^T \left(\Sigma^{-1}\right)_{xz}{}^T$, we have

$$\begin{aligned}
P(x,z)\sum_x P(y \mid z,x)P(x) &= (2\pi)^{-\frac{n}{2}} \left(\rho_{xx} (\det\Sigma) \left(\left(\Sigma^{-1}\right)_{xx} - \left(\Sigma_1^{-1}\right)_{xx} + \rho_{xx}{}^{-1} \right) \right)^{-\frac{1}{2}} \cdot \\
& \exp\left\{ -\frac{1}{2}(x-\mu_x)\left(\Sigma_1^{-1}\right)_{xx}(x-\mu_x) - (x-\mu_x)\cdot 0 \cdot (y-\mu_y) - (x-\mu_x)\left(\Sigma_1^{-1}\right)_{xz}{}^T (z-\mu_z) \right. \\
& -(y-\mu_y)\left[-\left(\Sigma^{-1}\right)_{xy} \left(\left(\Sigma^{-1}\right)_{xx} - \left(\Sigma_1^{-1}\right)_{xx} + \rho_{xx}{}^{-1} \right)^{-1} \left(\left(\Sigma^{-1}\right)_{xz} - \left(\Sigma_1^{-1}\right)_{xz} \right) + \left(\Sigma^{-1}\right)_{yz} \right] (z-\mu_z) \\
& -\frac{1}{2}(y-\mu_y)\left[-\left(\Sigma^{-1}\right)_{xy} \left(\left(\Sigma^{-1}\right)_{xx} - \left(\Sigma_1^{-1}\right)_{xx} + \rho_{xx}{}^{-1} \right)^{-1} \left(\Sigma^{-1}\right)_{xy} + \left(\Sigma^{-1}\right)_{yy} \right] (y-\mu_y) \\
& -\frac{1}{2}(z-\mu_z)^T \left[-\left(\left(\Sigma^{-1}\right)_{xx} - \left(\Sigma_1^{-1}\right)_{xx} + \rho_{xx}{}^{-1} \right)^{-1} \left(\left(\Sigma^{-1}\right)_{xz}{}^T - \left(\Sigma_1^{-1}\right)_{xz}{}^T \right) \left(\left(\Sigma^{-1}\right)_{xz} - \left(\Sigma_1^{-1}\right)_{xz} \right) + \left(\Sigma^{-1}\right)_{zz} - \left(\Sigma_1^{-1}\right)_{zz} + \left(\Sigma_1^{-1}\right)_{zz} \right] (z-\mu_z) \\
&= (2\pi)^{-\frac{n}{2}} \left(\rho_{xx} (\det\Sigma) \left(\left(\Sigma^{-1}\right)_{xx} - \left(\Sigma_1^{-1}\right)_{xx} + \rho_{xx}{}^{-1} \right) \right)^{-\frac{1}{2}} \cdot \exp\left\{ -\frac{1}{2}(u-\mu_u)^T C^{-1} (u-\mu_u) \right\}
\end{aligned} \qquad \text{(S13)}$$

where

$$C^{-1} = \begin{pmatrix} C_{xx} & C_{xy} & C_{xz} \\ C_{xy} & C_{yy} & C_{yz} \\ C_{xz}{}^T & C_{yz}{}^T & C_{zz} \end{pmatrix},$$

$$C_{xx} = \left(\Sigma_1^{-1}\right)_{xx}, C_{xy} = 0, C_{xz} = \left(\Sigma_1^{-1}\right)_{xz},$$

$$C_{yz} = \left(\Sigma^{-1}\right)_{yz} - \left(\Sigma^{-1}\right)_{xy} \left(\left(\Sigma^{-1}\right)_{xx} - \left(\Sigma_1^{-1}\right)_{xx} + \rho_{xx}{}^{-1} \right)^{-1} \left(\left(\Sigma^{-1}\right)_{xz} - \left(\Sigma_1^{-1}\right)_{xz} \right),$$

$$C_{yy} = \left(\Sigma^{-1}\right)_{yy} - \left(\Sigma^{-1}\right)_{xy}^2 \left(\left(\Sigma^{-1}\right)_{xx} - \left(\Sigma_1^{-1}\right)_{xx} + \rho_{xx}{}^{-1} \right)^{-1},$$

$$C_{zz} = \left(\Sigma^{-1}\right)_{zz} - \left(\left(\Sigma^{-1}\right)_{xx} - \left(\Sigma_1^{-1}\right)_{xx} + \rho_{xx}{}^{-1} \right)^{-1} \left(\left(\Sigma^{-1}\right)_{xz}{}^T - \left(\Sigma_1^{-1}\right)_{xz}{}^T \right) \left(\left(\Sigma^{-1}\right)_{xz} - \left(\Sigma_1^{-1}\right)_{xz} \right).$$

So we can get

$$\begin{aligned}
P(x,z)\sum_x P(y \mid z,x)P(x) &= (2\pi)^{-\frac{n}{2}} \left(\rho_{xx} \left(\left(\Sigma^{-1}\right)_{xx} - \left(\Sigma_1^{-1}\right)_{xx} + \rho_{xx}{}^{-1} \right) \det\Sigma \right)^{-\frac{1}{2}} \cdot \exp\left\{ -\frac{1}{2}(u-\mu_u)^T C^{-1} (u-\mu_u) \right\} \\
&= \left(\rho_{xx} \left(\left(\Sigma^{-1}\right)_{xx} - \left(\Sigma_1^{-1}\right)_{xx} + \rho_{xx}{}^{-1} \right) \right)^{-\frac{1}{2}} \left(\frac{\det C}{\det\Sigma} \right)^{\frac{1}{2}} \cdot (2\pi)^{-\frac{n}{2}} (\det C)^{-\frac{1}{2}} \cdot \exp\left\{ -\frac{1}{2}(u-\mu_u)^T C^{-1} (u-\mu_u) \right\} \\
&= C_1 \cdot Q(x,y,z),
\end{aligned} \qquad \text{(S14)}$$

where

$$C_1 = \left(\rho_{xx}\left(\left(\Sigma^{-1}\right)_{xx} - \left(\Sigma_1^{-1}\right)_{xx} + \rho_{xx}{}^{-1}\right)\right)^{-\frac{1}{2}} \left(\frac{\det C}{\det \Sigma}\right)^{\frac{1}{2}},$$

$$Q(x,y,z) = (2\pi)^{-\frac{n}{2}} (\det C)^{-\frac{1}{2}} \cdot \exp\left\{-\frac{1}{2}(u-\mu_u)^T C^{-1}(u-\mu_u)\right\}.$$

So the KL-divergence equals to

$$\begin{aligned} D_{KL}(P(X,Y,Z) \,\|\, P_{X\to Y}(X,Y,Z)) &= \sum_{x,y,z} P(x,y,z) \ln \frac{P(x,y,z)}{C_1 \cdot Q(x,y,z)} \\ &= \sum_{x,y,z} P(x,y,z) \ln \frac{P(x,y,z)}{Q(x,y,z)} - \ln C_1 \\ &= D_{KL}(P(x,y,z) \,\|\, Q(x,y,z)) - \ln C_1. \end{aligned} \quad \text{(S15)}$$

The K-L divergence between two multivariate Gaussian distributions $p(x) = N(x;\mu_p,\Sigma_p)$ and $q(x) = N(x;\mu_q,\Sigma_q)$ can also be written as(9)

$$D_{KL}(p \,\|\, q) = \frac{1}{2}\left(tr(\Sigma_q^{-1}\Sigma_p) + (\mu_p - \mu_q)^T \Sigma_q^{-1}(\mu_p - \mu_q) - \ln\left(\frac{\det \Sigma_p}{\det \Sigma_q}\right) - n \right). \quad \text{(S16)}$$

With Eqs(S15) and (S16), we have

$$D_{KL}(P \,\|\, P_{X\to Y}) = \frac{1}{2}\left(tr(C^{-1}\Sigma) - \ln\left(\frac{\det \Sigma}{\det C}\right) - n \right) - \ln C_1,$$

which can be rewrited as

$$D_{KL}(P \,\|\, P_{X\to Y}) = \frac{1}{2}\left(tr(C^{-1}\Sigma) + \ln C_0 - n \right),$$

where

$$C_0 = \rho_{xx}\left(\left(\Sigma^{-1}\right)_{xx} - \left(\Sigma_1^{-1}\right)_{xx} + \rho_{xx}{}^{-1}\right).$$

Similarly, we have the equation $D_{KL}(P \,\|\, P_{Y\to X}) = \frac{1}{2}\left(tr(\tilde{C}^{-1}\tilde{\Sigma}) + \ln \tilde{C}_0 - n \right)$, $\tilde{C}_0 = \rho_{yy}\left(\left(\tilde{\Sigma}^{-1}\right)_{yy} - \left(\tilde{\Sigma}_1^{-1}\right)_{yy} + \rho_{yy}{}^{-1}\right)$.

With above equations, we will have the result of Eq(S10).

Theorem 1 provided a simple computation formula of CMI2. With the general hypothesis of Gaussian distribution underlying gene expression data, CMI2 between a pair of genes can be computed by a concise formula involving covariance matrices and Pearson correlation coefficient of the related gene expression profiles.

5. Enrichment analysis of NRSF target genes with cancer gene annotations

NRSF target genes (39)

STAT5B, MAP2K2, MAZ, CEBPB, E2F3, BCLAF1, TP53, ZBTB7A, STAT3, PU1, SHC1, YY1, NFKB1, Max, TGFBR1, CBL, SOS2, STAT5A, NFKBIA, HRAS, HDAC1, MYC, PIK3CA, NRAS, RB1, RPS6KB1, RFX5, SMAD4, MTOR, BAD, CCNT2, E2F1, Egr-1, CDK4, CDK6, CHUK, CRKL, CTBP1, ELF1.

Table S1. Enrichment analysis results

No.	Base Pathways[a]	Pathway (Database)	Genes in pathway	Genes overlapped	p-value[b]	q-value[c]
1	ALL	CHRONIC MYELOID LEUKEMIA (KEGG)	73	18	2.94e-29	2.45e-26
2	ALL	ACUTE MYELOID LEUKEMIA (KEGG)	60	15	3.32e-24	1.38e-21
3	ALL	PATHWAYS IN CANCER (KEGG)	328	22	1.02e-23	2.83e-21
4	ALL	PROSTATE CANCER (KEGG)	89	16	2.17e-23	4.53e-21
5	ALL	ENDOMETRIAL CANCER (KEGG)	52	12	8.22e-19	1.37e-16
6	ALL	COLORECTAL CANCER (KEGG)	62	12	8.25e-18	1.15e-15
7	ALL	GLIOMA (KEGG)	65	12	1.52e-17	1.81e-15
8	ALL	PANCREATIC CANCER (KEGG)	70	12	3.93e-17	4.09e-15
9	ALL	NON SMALL CELL LUNG CANCER (KEGG)	54	11	1.34e-16	1.24e-14
10	ALL	ERBB SIGNALING PATHWAY (KEGG)	87	11	3.40e-14	2.84e-12
11	ALL	SMALL CELL LUNG CANCER (KEGG)	84	12	3.21e-14	2.43e-12
12	ALL	MELANOMA (KEGG)	71	11	1.75e-13	1.22e-11
13	ALL	BLADDER CANCER (KEGG)	42	9	1.64e-12	1.05e-10
14	ALL	DOWN STREAM SIGNAL TRANSDUCTION (REACTOME)	35	8	1.83e-11	1.09e-9
15	ALL	CTCF PATHWAY (BIOCARTA)	23	7	4.01e-11	2.09e-9

a) Pathway set used for the test. CGC: 146 Cancer Gene Censers gene-based pathways, CGI: 179 Cancer Gene Index gene-based pathways, and ALL: All 833 pathways from BioCarta/KEGG/Reactome databases.

b) p-value from Fisher's exact test for the overlapping gene.

c) q-value for false discovery rate control.

6. Enrichment analysis of BCLAF1 targeted genes with cancer gene annotations

BCLAF1 target genes (35)

PHF8, AKT2, ZBTB7A, YY1, Max, CHD1, TGFBR1, SOS2, TCF7L2, HRAS, HDAC1, MYC, MAPK3, PIK3CA, NRAS, PML, PIK3R1, RAF1, PPARD, RB1, RARA, RPS6KB1, RFX5, SMAD4, SMAD3, BAD, CCNT2 , NRSF, BRAF, CDK4, CDK6, CEBPA, CHUK, CRK, CRKL.

Table S2. Enrichment analysis results

No.	Base Pathways[a]	Pathway (Database)	Genes in pathway	Genes overlapped	p-value[b]	q-value[c]
1	ALL	CHRONIC MYELOID LEUKEMIA (KEGG)	73	22	2.90e-35	2.41e-32
2	ALL	PATHWAYS IN CANCER (KEGG)	328	28	5.05e-30	2.10e-27
3	ALL	ACUTE MYELOID LEUKEMIA (KEGG)	60	18	1.94e-28	5.38e-26
4	ALL	PANCREATIC CANCER (KEGG)	70	14	3.03e-19	6.30e-17
5	ALL	NON SMALL CELL LUNG CANCER (KEGG)	54	13	5.07e-19	8.45e-17
6	ALL	ERBB SIGNALING PATHWAY (KEGG)	87	14	7.74e-18	1.07e-15
7	ALL	ENDOMETRIAL CANCER (KEGG)	52	12	2.46e-17	2.93e-15
8	ALL	COLORECTAL CANCER (KEGG)	62	12	2.44e-16	2.55e-14
9	ALL	GLIOMA (KEGG)	65	12	4.49e-16	4.15e-14
10	ALL	PROSTATE CANCER (KEGG)	89	13	5.34e-16	4.45e-14
11	ALL	MELANOMA (KEGG)	71	12	1.38e-15	1.05e-13
12	ALL	RACCYCD PATHWAY (BIOCARTA)	26	9	5.89e-15	4.09e-13
13	ALL	RENAL CELL CARCINOMA (KEGG)	70	11	5.83e-14	3.74e-12
14	ALL	INSULIN SIGNALING PATHWAY (KEGG)	137	13	1.57e-13	9.37e-12
15	ALL	GLEEVEC PATHWAY (BIOCARTA)	23	8	2.10e-13	1.16e-11
16	ALL	IL2RB PATHWAY (BIOCARTA)	38	9	2.89e-13	1.50e-11
17	ALL	NEUROTROPHIN SIGNALING PATHWAY (KEGG)	126	12	1.57e-12	7.70e-11
18	ALL	TRKA SIGNALLING FROM THE PLASMA MEMBRANE(REACTOME)	103	11	4.52e-12	2.09e-10
19	ALL	DOWN STREAM SIGNAL TRANSDUCTION (REACTOME)	35	8	9.47e-12	4.15e-10
20	ALL	RAS PATHWAY (BIOCARTA)	23	7	2.27e-11	9.40e-10

References

1. Faith, J.J., Hayete, B., Thaden, J.T., Mogno, I., Wierzbowski, J., Cottarel, G., Kasif, S., Collins, J.J. and Gardner, T.S. (2007) Large-scale mapping and validation of Escherichia coli transcriptional regulation from a compendium of expression profiles. *PLoS biology*, **5**, e8.
2. Treviño III, S., Sun, Y., Cooper, T.F. and Bassler, K.E. (2012) Robust detection of hierarchical communities from Escherichia coli gene expression data. *PLoS computational biology*, **8**, e1002391.
3. Frenzel, S. and Pompe, B. (2007) Partial mutual information for coupling analysis of multivariate time series. *Physical review letters*, **99**, 204101.
4. Zhang, X., Zhao, X.M., He, K., Lu, L., Cao, Y., Liu, J., Hao, J.K., Liu, Z.P. and Chen, L. (2012) Inferring gene regulatory networks from gene expression data by path consistency algorithm based on conditional mutual information. *Bioinformatics*, **28**, 98-104.
5. Reshef, D.N., Reshef, Y.A., Finucane, H.K., Grossman, S.R., McVean, G., Turnbaugh, P.J., Lander, E.S., Mitzenmacher, M. and Sabeti, P.C. (2011) Detecting novel associations in large data sets. *Science*, **334**, 1518-1524.
6. Janzing, D., Balduzzi, D., Grosse-Wentrup, M. and Schoelkopf, B. (2012) Quantifying causal influences. *Ann. Statist.*, **41**, 2324-2358.
7. Runge, J., Heitzig, J., Marwan, N. and Kurths, J. (2012) Quantifying causal coupling strength: a lag-specific measure for multivariate time series related to transfer entropy. *Physical review. E, Statistical, nonlinear, and soft matter physics*, **86**, 061121.
8. Kullback, S. and Leibler, R.A. (1951) On information and sufficiency. *The Annals of Mathematical Statistics*, **22**, 79-86.
9. Roberts, S.J. and Penny, W.D. (2002) Variational Bayes for generalized autoregressive models. *Signal Processing, IEEE Transactions on*, **50**, 2245-2257.
